# The Activity of Plant-Derived Ren’s Oligopeptides-1 against the Pseudorabies Virus

**DOI:** 10.3390/ani12111341

**Published:** 2022-05-25

**Authors:** Danmei Xiao, Yu He, Qin Xiao, Luxia Cai, Haoqi Wang, Aikebaier Reheman, Ke Xiao

**Affiliations:** 1 College of Veterinary Medicine, Huazhong Agricultural University, Wuhan 430070, China; xdm2108@126.com (D.X.); jz4607386@163.com (Y.H.); maimu220@163.com (Q.X.); luxia_cai@163.com (L.C.); zyx960523@163.com (H.W.); 2 College of Animal Science and Technology, Tarim University, Alar 843300, China; 3 State Key Laboratory of Agricultural Microbiology, Huazhong Agricultural University, Wuhan 430070, China

**Keywords:** Ren’s oligopeptides-1, pseudorabies virus (PRV), high content screening, RTCA

## Abstract

**Simple Summary:**

Although artificial antiviral drugs can effectively inhibit the proliferation of viruses, these drugs not only have serious side effects, but also create drug-resistant virus strains and produce harmful drug residues. In comparison, natural antiviral compounds have many distinctive advantages. For example, natural compounds, in general, have a lower toxicity, lower price, and better antiviral effects. Nowadays, wide applications of natural antiviral compounds not only provide us with safe meat products, but also reduce the abuse of antibiotics, thus reducing the number of drug-resilient microbes and viruses. The purpose of this study was to demonstrate the antiviral activity of Ren’s oligopeptides-1 against the pseudorabies virus (PRV). Ren’s oligopeptides-1 is a plant-derived drug with an antiviral activity against herpesvirus. Ren’s oligopeptides-1 contains ginger, garlic, onion, banana peel, and bitter melon. We discovered that the CC_50_ value of Ren’s oligopeptides-1 was 15 mg/mL, while the EC_50_ value was 6 mg/mL. This indicated that the concentration of 6 mg/mL was optimal for inhibiting the replication of PRV. In this study, PRV was used to conduct in vitro experiments on PK15 cells to explore the antiviral effect of Ren’s oligopeptides-1, which could become a promising feed additive.

**Abstract:**

Newly synthesized Ren’s oligopeptides-1 was found to have an antiviral effect in clinical trials, and the purpose of this study was to further demonstrate the antiviral activity of Ren’s oligopeptides-1 against the PRV 152-GFP strain. We used the real-time cell analysis system (RTCA) to detect the cytotoxicity of different concentrations of Ren’s oligopeptides-1. We then applied high content screening (HCS) to detect the antiviral activity of Ren’s oligopeptides-1 against PRV. Meanwhile, the fluorescence signal of the virus was collected in real time and the expression levels of the related genes in the PK15 cells infected with PRV were detected using real-time PCR. At the mRNA level, we discovered that, at a concentration of 6 mg/mL, Ren’s oligopeptides-1 reduced the expression of pseudorabies virus (PRV) genes such as IE180, UL18, UL54, and UL21 at a concentration of 6 mg/mL. We then determined that Ren’s oligopeptides-1 has an EC_50_ value of 6 mg/mL, and at this level, no cytotoxicity was observed.

## 1. Introduction

Pseudorabies (PR) is still a serious epidemic, especially for the livestock industry around the globe. Hungarian veterinarian Aujeszky was the first to prove that the causative agent of pseudorabies is the pseudorabies virus (PRV) [1]. PRV is a double-stranded DNA herpes virus that is neurotropic and can induce various diseases in animals [2]. The only natural host for PRV is pigs, but it can be transmitted to birds and other mammals [3]. Aujeszky’s disease in wild swine appears to be a source of infection for domestic pigs, as well as domestic and wild carnivores [4]. PRV is mainly spread via direct contact, but may also be transmitted by air, water, and contaminated fomites. PRV is extremely harmful to pigs and has caused serious losses to the world’s pig industry. In China, pigs were first reported to be infected with PRV in 1950 [5]. In China, the overall epidemiological trend of PRV is not optimistic—currently, the average rate of PRV infection exceeds 10.0% in the eastern and central parts of China, while being slightly lower than 7.0% in the northwestern region [6]. In Shandong Province, China, the average rate of PRV infection in pigs was 11.8% in 2015, 2017, and 2018 [7]. In Tianjin, China, blood samples were collected from 228 pig farms over the time span of 2010 to 2018 [8]. The final results showed that 46.70% of the serum samples tested positive for PRV infection. About 20% to 45% of pigs are infected with PRV, indicating that the virus is characterized by a high prevalence and variability.

PRV is highly contagious and infected pigs shed large quantities of the virus in secretions and excretions [9], rendering the prevention and control of the virus through vaccination ineffective [10]. The first live genetically modified vaccine was imported from Hungary in the 1970s [11]. However, PR outbreaks have persisted in farms vaccinated with Bartha-K61. The PR outbreak is mainly due to mutations in the PRV genome, which explains the low efficacy of the traditional Bartha-K61 vaccine [12]. Subsequently, new genetically modified engineered vaccines were created, including a PRV-based gE/gI/TK deletion vaccine [13] and a PRV-based gE/gI deletion vaccine [14]. The herpes virus in pigs has the characteristics of fast transmission and high lethality. All of these properties bring great difficulties to the research and development as well as clinical application of pig anti-herpes virus vaccines.

As a result, scientists have been shifting their focus to the development of anti-herpes virus drugs. In general, there are four types of anti-herpes virus drugs, including nucleoside anti-herpes virus drugs, non-nucleoside anti-herpes virus drugs, plant-derived anti-herpes virus drugs [15], and new anti-herpes virus drugs [16]. Acyclovir is currently the drug of choice against the herpes virus [17,18,19]. Non-nucleoside antiviral drugs are often used in patients who are resistant or allergic to nucleoside drugs [20]. The novel anti-herpes virus drug inhibits viral replication by inhibiting viral DNA helicase [21]. After Ribavirin and Acyclovir were put into clinical use, there were reports of drug-resistant strains as well as the shortcomings of their drug residues. Synthesized chemical antiviral drugs have great toxic side effects on host cells. Therefore, it is of great significance to dig out antiviral drugs from economical plant resources. For example, polysaccharides isolated from the extract of *Radix isatidis* have antiviral effects, and different concentrations of *Radix Isatidis* polysaccharides can all inhibit the replication of PRV [22]. *Prunella vulgaris* polysaccharides can inhibit the proliferation of the herpes virus by inhibiting the adsorption and entry of the virus [23]. *Astragalus* polysaccharide can inhibit the replication of PRV [24]. Monomer compounds from a variety of plants, such as Geranium wilfordii, Anemarrhena, Glycyrrhizin, and Cepharanthine, have been reported to have anti-herpes simplex virus effects [25]. *Gardenia* extracts T9 and TH can competitively inhibit the binding site of virus and host cells, and increase the expression of the IFN-γ gene in host cells, thereby reducing the replication of herpes simplex virus I [26].

With the improvement in the ability to extract and analyze plant active ingredients, drugs with an anti-herpes virus effect from plants have become a research hotspot. Plant-derived drugs have few side effects, lower drug resistance, lower price, many drug targets, and immunomodulatory functions. In this study, PRV was used to conduct in vitro experiments in PK15 cells (ATCC^R^ CCL-33) so as to explore the antiviral effect of Ren’s oligopeptides-1 and to provide a new reference for the development of PRV antiviral drugs.

## 2. Materials and Methods

### 2.1. Natural Extract Preparation

**Figure 1:**
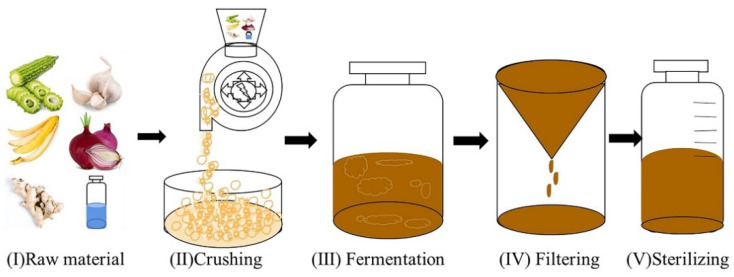
Drug preparation flow chart of Ren’s oligopeptides-1.

The extraction process of the drug is as follows (Figure 1). Ren’s oligopeptides-1 [27,28,29,30] contains ginger (20%), garlic (20%), onion (20%), banana peel (10%), and bitter melon (20%), all of which were obtained from Wuhan Shijie Maide Biotechnology Company Limited. The first step in the preparation of the natural extract was to crush the bulk raw material into powder. The crushed particle size of the raw material was 355 µm. ddH_2_O was then added to the mixture, which was kept under stirring for 8 days. The weight ratio of amylase to the mixture was 1:10,000. Then, the amylase was put into the mixture for enzymatic hydrolysis, and the enzymatic hydrolysis time was 8 days. Finally, the supernatant was collected for disinfection. After boiling to sterilize, Ren’s oligopeptides-1 was stored at 4 °C for later use. The concentration of Ren’s oligopeptides-1 extracted from the plant was 900 mg/mL. In the subsequent experiments, the drug was eluted in DMEM. 

### 2.2. Cell Line Culture

PK15 cells (ATCC^R^ CCL-33) were grown in 25 cm^2^ culture flasks containing Dulbecco’s Modified Eagle’s Medium (DMEM) supplemented with 10% fetal bovine serum (FBS, Gibco), 50 units/mL penicillin, and 50 µg/mL streptomycin (Gibco). The flasks were stored at 37 °C in an incubator, which provided a humidified atmosphere containing 5% CO_2_. When the PK15 cells grew to cover 90% of the flasks, the cells were subcultured in fresh medium. The subculturing process was repeated three times a week.

### 2.3. TCID_50_ Assay

The PRV 152-GFP strain was used in this study (a gift from K. Xiao, Huazhong Agricultural University). PK15 cells (ATCC^R^ CCL-33) were inoculated in a 25 cm^2^ culture flask, and PRV 152-GFP virus strain dilution was added when the cell proliferation was about 80%. The number of fluorescent cells was observed under a fluorescence microscope when 90% of the cells in the 25 cm^2^ culture flask showed green fluorescence. The virus-containing material was then collected, freeze-thawed three times in liquid nitrogen, and centrifuged at 10,000× *g* for 15 min at 4 °C. PK15 cells at 2 × 10^5^ cells/mL were seeded in 96-well microplates overnight. Serial 10-fold dilutions (from 10^−1^ to 10^−10^) of the original PRV 152-GFP virus were made with DMEM using a culture medium in centrifuge tubes. Different dilutions of the virus were inoculated into 96-well culture plates, and each dilution was inoculated in a vertical row, and a total of 8 wells were repeated. The 12th row was the control group. Each well was inoculated with 100 µL dilutions. The cytopathic effect (CPE) was recorded and observed every day, and the virus titer was counted using the Reed−Muench method [31].

### 2.4. Cytotoxic Assays

The detection of the drug toxicity was performed using a real-time cell analysis system (RTCA), which enabled tracking of the cell growth during experiments [32]. When the PK15 cells in the 25 cm^2^ culture flask grew to 80–90% confluent, 1 mL of trypsin was added to the culture flask and allowed to stand for 5 min at room temperature. Then, the cell solution was collected in a 15 mL centrifuge tube and centrifuged at 1500× *g* for 5 min. Afterward, 2 mL of 1× PBS solution was added to the cell pellet and the mixture was centrifuged at 1500× *g* for 5 min. Finally, the cells were resuspended in 1 mL of DMEM medium containing 2% FBS. 

The cell solution (10 µL) was used for cell counting, ensuring that the concentration of cells in the RTCA experiment was at least 1 × 10^5^ cells/mL. DMEM was used as a negative control, and 1 × 10^4^ cells/100 µL PK15 cells were used as a positive control group. The control groups were the cells without treatment. Firstly, 50 μL of DMEM with 2% FBS was added to each well of the E-plate 16, and then 100 μL of the cell suspension was added to the drug groups with different concentrations and a positive control group. After 12 h, the E-plate 16 was removed from the RTCA machine, and different concentrations of Ren’s oligopeptides-1 were added at 3 mg/mL, 6 mg/mL, 15 mg/mL, and 30 mg/mL. Three biological replicates were set up for each concentration. E-plate 16 was placed into the RTCA instrument. The above steps were repeated for drug concentrations of 3 mg/mL, 6 mg/mL, 15 mg/mL, and 30 mg/mL. The statistical method software (xCELLigence RTCA DP) is part of the real-time cell analysis system (RTCA).

### 2.5. High-Content Screening of Ren’s Oligopeptides-1 against PRV

High-content system compound screening was used to observe the activity of the natural extracts against PRV and to exploit the GFP signal. A combination of optical microscope technology and a computer analysis system was also used [33,34]. The cells were seeded into 96-well microplates at a density of 1 × 10^4^ cells/150 µL, and were cultured overnight in a 37 °C, 5% CO_2_ incubator. A control group (infected cells without treatment) and four experimental groups (with the virus and different concentrations of drugs) were designed, with three replicates for each group. PK15 cells were infected in vitro with the PRV 152-GFP strain at an MOI of 4 for 1 h, and the drugs were added at a concentration of 3 mg/mL, 6 mg/mL, 15 mg/mL, and 30 mg/mL. After 3 h, the cells were changed to a new DMEM medium, and the unadsorbed viruses were discarded. In the high-content analysis system, four fields of view were randomly selected for each well, and then the amount of virus fluorescence in the field of view was collected in real time and plotted with GraphPad Prism 6. The statistical method software (Opera phenix, HarmonyR4.9) is part of the high-content screening system.

### 2.6. Quantitation of PRV Replication by Real-Time PCR

First, 2 × 10^6^/600 µL PK15 cells were inoculated into 24-well microplates and placed in a 37 °C, 5%CO_2_ incubator overnight. The PK15 cells (ATCC^R^ CCL-33) in the 24-well microplates were washed with 1 × PBS, and two experimental groups and two control groups were set up. In vitro infection of PK15 cells was conducted with the PRV 152-GFP strain at an MOI of 4. In the first group, PK15 cells were infected for 3 h by adding the PRV 152-GFP strain after adding Ren’s oligopeptides-1 at a concentration of 6 mg/mL for 9 h, and the control group was replaced by DMEM. In the second group, PK15 cells were infected for 6 h in vitro by adding the PRV 152-GFP strain after adding the drug at a concentration of 6 mg/mL for 6 h, and the control group was replaced by DMEM. The cells were then harvested for RNA isolation and qPCR as described previously (Table 1). The relative mRNA expression was calculated using the 2^−^^ΔΔCt^ method with Swine-29 as an internal reference gene. Both groups of experiments used the RNAiso Plus reagent to purify and collect the viruses in the PK15 cells [35]. After DNase treatment, reverse transcription was performed using the ReverTra Ace qPCR RT Master Mix kit (TOYOBO, Shanghai, China). The qPCR primer sequences were designed according to Genebank [36]. 

## 3. Results

### 3.1. Cytotoxicity of the Drug Ren’s Oligopeptides-1

**Figure 2:**
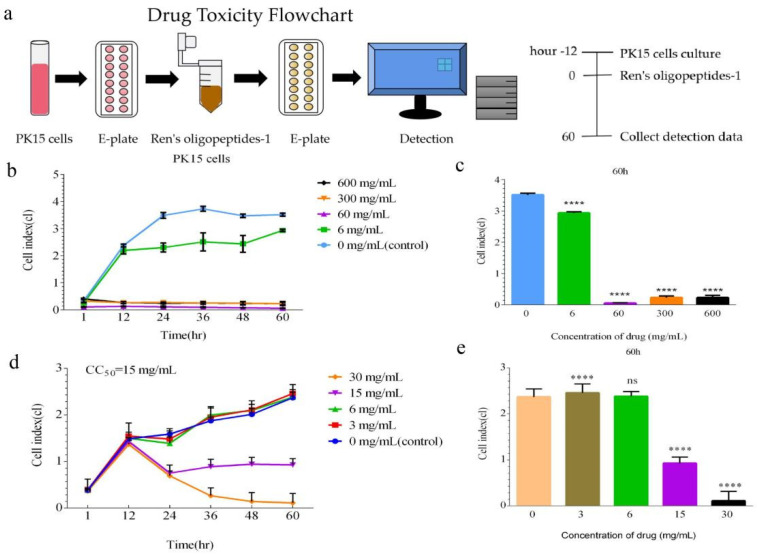
Detection of the cytotoxicity of Ren’s oligopeptides-1 at different concentrations. (**a**) Flow chart of drug cytotoxicity assay using the real-time cell analysis (RTCA) method. (**b**) Cell index plots for 6 mg/mL, 60 mg/mL, 300 mg/mL, and 600 mg/mL drug concentrations. (**c**) Significant differences in the cell index of each group at 6 mg/mL, 60 mg/mL, 300 mg/mL, and 600 mg/mL drug concentrations at the 60 h. (**d**) Cell index plots for drug concentrations of 3 mg/mL, 6 mg/mL, 15 mg/mL, and 30 mg/mL. (**e**) Significant differences in the cell index of each group at the 60 h for drug concentrations of 3 mg/mL, 6 mg/mL, 15 mg/mL, and 30 mg/mL. ns, not significant (*p* > 0.05) and **** *p* < 0.0001 from the Student’s *t*-test. Error bars represent the SD from three independent experiments.

Determining the toxicity of Ren’s oligopeptides-1 to PK15 cells was a prerequisite for all other studies. We observed that only the cells in the 6 mg/mL treatment group grew, and the cells in the other three groups showed no sign of growth (Figure 2b). The significance of the four experimental groups compared with the control group was less than 0.0001 (Figure 2c). According to the results (Figure 2d), the cell proliferation indexes of the treatment group at concentrations of 3 mg/mL and 6 mg/mL were roughly the same as those of the control group, and the drug had no inhibitory effect on cell proliferation at these concentrations. The significance of the 3 mg/mL treatment group and the control group was less than 0.0001. There was no significant difference between the 6 mg/mL treatment group and the control group (Figure 2e). In the treatment group with a concentration of 15 mg/mL, the number of cells decreased by half after adding the drug for 12 h, and then remained stable. The number of cells in the 30 mg/mL drug group gradually decreased to zero, indicating that this concentration of drug was toxic to the cells.

### 3.2. The Effect of the Drug Ren’s Oligopeptides-1 on Viral Replication

**Figure 3:**
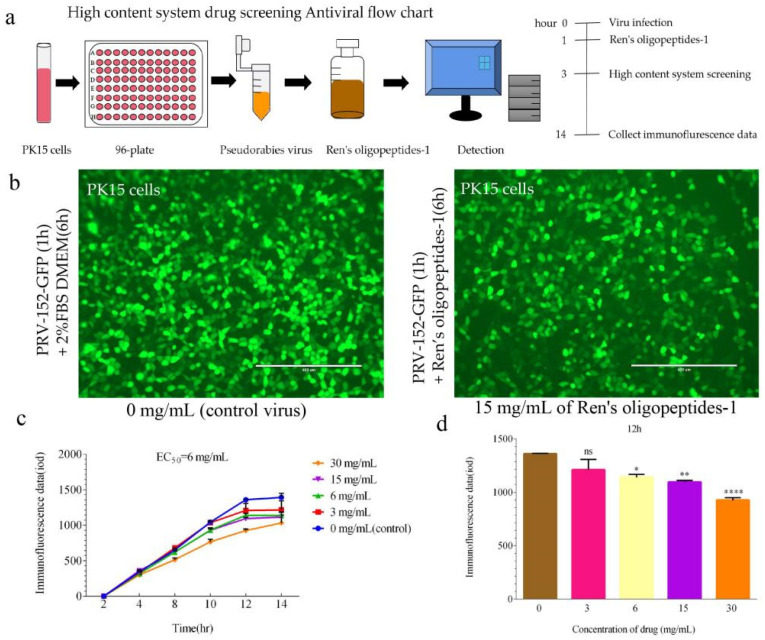
Ren’s oligopeptides-1 inhibits PRV-associated replication in PK15 cells. (**a**) High-content system drug screening antiviral flow chart. (**b**) Infected cells’ immunofluorescence plots were treated with 0 mg/mL and 15 mg/mL of Ren’s oligopeptides-1. (**c**) Infected cells were treated with 3 mg/mL, 6 mg/mL, 15 mg/mL, and 30 mg/mL of Ren’s oligopeptides-1; untreated and infected cells (PK15). A nonlinear regression analysis utilizing these data determined the EC_50_ for Ren’s oligopeptides-1 to be 6 mg/mL. (**d**) Significant differences in the immunofluorescence signal of each group at the 12 h for 3 mg/mL, 6 mg/mL, 15 mg/mL, and 30 mg/mL of Ren’s oligopeptides-1. ns, not significant (*p* > 0.05), * *p* < 0.05, ** *p* < 0.01, and **** *p* < 0.0001 from the Student’s *t*-test. Error bars represent the SD from five independent experiments.

Within the safe concentration range, the immunofluorescence signals of the 6 mg/mL treatment group and the 15 mg/mL treatment group were roughly the same (Figure 3b). Compared with the control group, the immunofluorescence signal of the treatment group with a concentration of 6 mg/mL decreased the most, and the *p*-value was less than 0.01. The signal of the virus fluorescence in the treatment was significantly lower than that of the control (Figure 3c). Compared with the control group in the four groups, the significant difference gradually increased (Figure 3d).

### 3.3. mRNA Expression Levels of PRV under the Influence of Inhibitors

**Figure 4:**
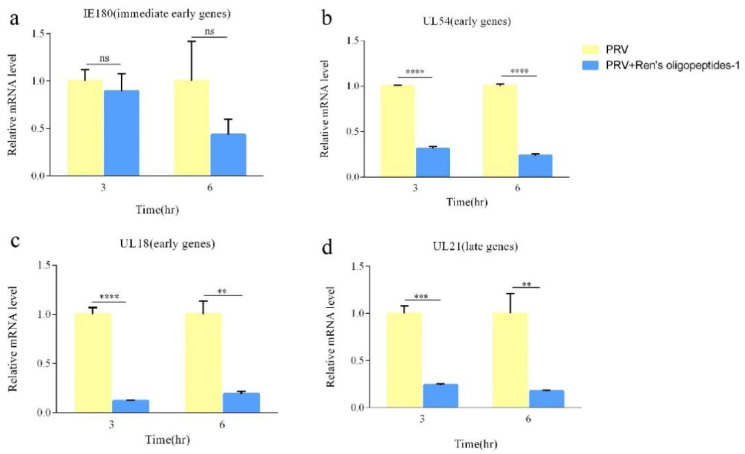
Viral RNA expression as measured by real-time PCR in PRV-infected PK15 cells treated with 6 mg/mL of Ren’s oligopeptides-1. (**a**) Expression levels of IE180 at 3 and 6 h following treatment with Ren’s oligopeptides-1. (**b**) Expression levels of UL54 at 3 and 6 h following treatment with Ren’s oligopeptides-1. (**c**) Expression levels of UL18 at 3 and 6 h following treatment with Ren’s oligopeptides-1. (**d**) Expression levels of UL21 at 3 and 6 h following treatment with Ren’s oligopeptides-1. ns, not significant (*p* > 0.05); ** *p* < 0.01, *** *p* < 0.001, and **** *p* < 0.0001 from the Student’s *t*-test. Error bars represent SD from three independent experiments.

To confirm the regulation of the PRV gene supporting a role for Ren’s oligopeptides-1 in the effect on viral replication, we analyzed the mRNA expression levels of PRV around several immediate early genes, early genes, and late genes (including IE180, UL54, UL18, and UL21). Next, PK15 cells were infected with PRV for different periods and then subjected to qPCR analysis. We found that Ren’s oligopeptides-1 could significantly decrease the expression of these genes at multiple time points after infection. As shown in Figure 4a–d, the mRNA expression levels of PRV were reduced following 9 and 6 h of treatment with Ren’s oligopeptides-1. The results showed that the expression levels of these four IE180, UL18, UL54, and UL21 genes were significantly inhibited by Ren’s oligopeptides-1.

## 4. Discussion

Viruses are one of the main causes of harm to human and animal health, including the pseudorabies virus (PRV). Natural antiviral compounds have many advantages compared with synthesized chemicals. For example, natural compounds have the characteristics of a lower toxicity, lower price, and better antiviral effects. Ren’s oligopeptide-1 is the extract of five natural plants, including ginger, garlic, onion, banana peel, and bitter melon. The drug can be derived from daily food, making it easy to obtain. Ren’s oligopeptide-1 has the merits of less residue, little drug resistance, fewer side effects, and lower cost. It can improve the animal health and growth performance of pigs and is expected to become a promising feed additive [37]. It is imperative to develop natural antiviral compounds that can provide new ideas for the treatment and prevention of viral diseases.

xCELLigence was used to monitor the cytotoxicity of drugs in real time [38], which reduced the time and cost. The inhibitory concentration of the compound on the cell growth at each time point was obtained through real-time xCELLigence analysis, and the compound concentration with the lowest cytotoxicity was selected for subsequent experiments. We found that the CC_50_ value of Ren’s oligopeptides-1 was 15 mg/mL. Ren’s oligopeptides-1 has the characteristics of a wide range of action and lower toxicity, which is beneficial to public health security and human economic development. 

Ren’s oligopeptides-1 can inhibit the replication of PRV, which is related to its plant extracts. PRV is neurotropic. Ren’s oligopeptides-1 contains ginger, which may have neuroprotective effects, because ginger contains several phenolic compounds, such as gingerol and shogaol. These chemicals have various biological activities, including antioxidant, anti-inflammatory, and antibacterial properties [39]. The bioactive compounds in ginger can modulate cell death or cell survival signaling molecules by ameliorating neurological symptoms and pathological conditions [40]. When the concentration of Ren’s oligopeptides-1 was 6 mg/mL, the expression of the IE180, UL18, UL54, and UL21 genes [41,42] of PRV was downregulated after both 3 h and 6 h of PRV infection. Moreover, Ren’s oligopeptides-1 can inhibit virus replication, and this ability could be related to the garlic. Studies have shown that garlic essential oil has an anti-coronavirus activity, mainly owing to the presence of allyl disulfide and allyl trisulfide. Garlic also has antioxidant, anti-inflammatory, antibacterial, antifungal, immunomodulatory, and neuroprotective properties [43,44]. The high-content system can directly observe the reduction in virus replication. It was found that, within the cell safety range, the lowest immunofluorescence signal was collected at a concentration of 6 mg/mL of Ren’s oligopeptides-1, demonstrating a reduction in viral replication. Furthermore, the replication of PRV was reduced under the influence of Ren’s oligopeptides-1. This property could be as a result of the onion. Again, studies have shown that onion phytochemicals show an anti-rabies activity mainly because of the presence of (+) catechins [45]. A kind of fructan was isolated from green onion as an anti-influenza virus substance (*Allium fistulosum* L.). Fructan does not show an anti-influenza virus activity in vitro, but has an inhibitory effect on virus in vivo when given orally to mice. The antiviral mechanism of polysaccharides appears to be dependent on the host’s immune system [46]. Both bitter melon and banana peels in Ren’s oligopeptides-1 have a strong biological activity. Bitter melon has an anti-inflammatory activity. Studies have shown that bitter melon extract can modulate inflammation-suppressing effects [47]. Bitter melon extract reduced the expression of LPS-induced inflammatory genes in Raw 264.7 cells, including the expression of IL-1α, IL-1β, and TNF-α [48]. Bitter melon also has anti-diabetic properties [49,50]. Banana peel is a good antioxidant [51]. Its extract is an important phenolic compound [52]. Ren’s oligopeptide-1 increases the total phenolic content and may have high antioxidant properties. Methanol and ethanol extracted from banana peels have an antibacterial potential [53]. Compared with other PRV antiviral drugs, Ren’s oligopeptide-1 can not only inhibit viral replication, but also has easy access to raw materials and a low drug cost. 

Ren’s oligopeptide-1 is a plant-derived anti-herpes virus drug with a wide range of actions and low toxicity. However, the safe dose and drug action time of Ren’s oligopeptide-1 need to be further explored and discussed. 

## 5. Conclusions

When it comes to reducing antibiotic abuse while having antiviral effects, Ren’s oligopeptides-1 offers a simple solution. It has a simple composition and is used as a feed additive. Ren’s oligopeptides-1 is inexpensive and readily available, ensuring safe meat products for humans. However, Ren’s oligopeptides-1 is still a long way off from clinical applications, and more experiments are needed to evaluate its economic aspects and efficacy in clinical conditions so that it can be used as a natural substitute for antibiotics in the swine industry.

## Figures and Tables

**Table 1:** qPCR primer sequences.

Gene	Forward Primer	Reverse Primer
swine-28S rRNA	5′-GGGCCGAAACGATCTCAACC-3′	5′-GCCGGGCTTCTTACCCATT-3′
PRV-UL54	5′-TGCAGCTACACCCTCGTCC-3′	5′-TCAAAACAGGTGGTTGCAGTAAA-3′
PRV-UL21	5′-TCAGCTGTTTCGGGCGC-3′	5′-ATTGAGGACGATGGAGATGTTGG-3′
PRV-UL18	5′-TGGTGCTGAACATGATCTTCC-3′	5′-GGATGAGCGACAGCAGGAT-3′
PRV-IE180	5′-CATCGTGCTGGACACCATCGAG-3′	5′-CATCGTGCTGGACACCATCGAG-3′

## Data Availability

Data supporting the reported results are contained within the article.
